# Simulation-Based Performance Assessment of Bulk Junctionless FET with Asymmetric Source/Drain for Ultrasensitive Detection of Biomolecules

**DOI:** 10.3390/bios15090597

**Published:** 2025-09-10

**Authors:** Jeongmin Son, M. Meyyappan, Kihyun Kim

**Affiliations:** 1 Division of Electronic and Information Engineering, Jeonbuk National University, Jeonju 54896, Republic of Korea; jungmin3173@jbnu.ac.kr; 2 Centre for Nanotechnology, Indian Institute of Technology Guwahati, Guwahati 781039, India; meyya@ieee.org; 3 Division of Electronic Engineering and Future Semiconductor Convergence Technology Research Center, Jeonbuk National University, Jeonju 54896, Republic of Korea

**Keywords:** biosensing, field effect transistor (FET), BioFET, TCAD simulation, asymmetric source and drain, field stop layer

## Abstract

Bio field-effect transistors (BioFETs) have attracted attention for their ability to rapidly detect physiological data with a simple structure. While conventional BioFETs offer high sensitivity, they often require reference electrodes or involve complex fabrication processes. A recently proposed bulk junctionless BioFET (Bulk JL-BioFET) features a simple fabrication process to address these issues. This structure utilizes a depletion region formed by a p-n junction, as the active layer is directly in contact with a substrate of the opposite type. As a result, the device can operate effectively with only two terminals—drain and source—without the need for a reference electrode. In this study, we propose a novel Bulk JL-BioFET, incorporating a doped field stop layer and an asymmetric source/drain structure, and verify its performance through simulations. The doped field stop layer blocks the electric field expansion, enhancing channel modulation, while the asymmetric source/drain structure promotes electron injection, reducing the on-off swing voltage and turn-on voltage. This improves the electrical performance, enabling lower power consumption and higher sensitivity. Simulation results show that the combination of these two novel features results in a sensitivity increase of approximately 30-fold. Moreover, high sensitivity was observed below the turn-on voltage region for all the structures when analyzing the sensitivity with overdrive voltage, identifying the optimal operating conditions. This study suggests that the combination of the doped field stop layer and asymmetric source/drain structure is an effective design strategy to maximize the sensing performance of BioFETs while minimizing power consumption.

## 1. Introduction

Biosensors are increasingly utilized in a wide range of applications, including point-of-care diagnostic systems [1], water quality monitoring [2], and wearable devices for smart technology [3]. Their growing demand is largely attributed to the capability of providing rapid and direct physiological measurements without the need for bulky or complex instrumentation. Recent advances have enabled the development of biosensors that meet essential performance criteria such as compact size, low power consumption, cost-effective fabrication, high sensitivity, and fast response times. Conventional laboratory-based analytical techniques, such as polymerase chain reaction (PCR) [4], enzyme-linked immunosorbent assay (ELISA) [5], and mass spectrometry [6], offer high sensitivity and specificity. However, their long analysis time, high equipment cost, and large size impose significant limitations on real-time or field-deployable applications.

The introduction of the ion-sensitive field-effect transistor (ISFET) in the early 1970s marked the emergence of a promising biosensor platform, owing to its real-time detection capability, compatibility with cost-effective CMOS fabrication process, and potential for large-scale integration [7]. Since the development of the ISFET technology, continuous advancements have led to the emergence of bio field-effect transistors (BioFETs), which achieve selectivity for specific analytes by functionalizing the gate insulator or metal with bioreceptors through various surface modification techniques [8]. Continuous research has since focused on optimizing channel materials and structural designs to further enhance the BioFET performance. A variety of channel materials, including silicon nanowire (SiNW) [9,10,11,12], carbon nanotubes (CNTs) [13,14], graphene [15], and MoS_2_ [16,17], have been explored. Among these candidates, SiNWs have been most extensively studied due to their high surface-to-volume ratio and excellent sensitivity. Several FET architectures have been proposed in parallel, such as dual-gate FET [18], nanosheet FET [19], and tunneling field-effect transistor (TFET) [20]. However, these structures often involve complex fabrication processes and increased power consumption, which pose challenges for practical biosensing applications.

To overcome the above challenges, our group previously proposed a bulk junctionless bio field-effect transistor (Bulk JL-BioFET) [21], which offers a simplified fabrication process and enables biosensing without the use of a reference electrode. Biosensing is achieved by monitoring changes in current, voltage, or resistance, which are induced by variations in the depletion region formed by a vertical p–n junction between the active layer and the substrate. These variations result from interactions between biomolecules and surface-bound bioreceptors. In the Bulk JL-BioFET, modulation of the depletion region thickness plays a critical role in determining device sensitivity. This modulation capability is strongly influenced by the doping concentrations in both the active layer and the substrate, necessitating precise control of these parameters. In the field of electronic devices, the incorporation of a doped field stop layer (DFSL) into bulk planar junctionless transistors has been shown to improve the on/off current ratio and reduce power consumption [22], which is also referred to as the pocket device. We adopt the DFSL concept for the first time in the context of a Bulk JL-BioFET in order to improve its sensitivity. In addition, an asymmetric source/drain (ASD) structure is implemented to further enhance the overall device performance. The electrical and biosensing performance of the proposed device, integrating both DFSL and ASD configurations, is analyzed using technology computer-aided design (TCAD) simulations to elucidate the underlying physical mechanisms. Key electrical parameters, including on-off swing voltage (V_swing_) and turn-on voltage (V_ON_), are evaluated, followed by a systematic investigation of the biomolecule detection capability.

## 2. Simulation Methods

### 2.1. Device Simulation Parameters

**Figure 1:**
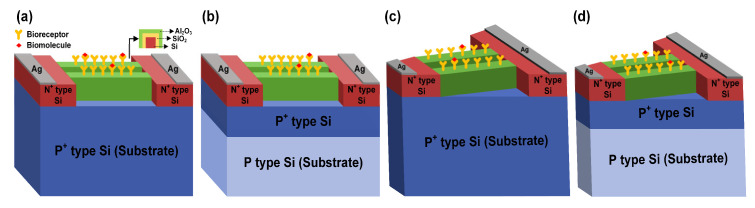
Schematic of various Bulk JL-BioFETs: (**a**) conventional Bulk JL-BioFET, (**b**) DFSL-Bulk JL-BioFET (**c**) ASD-Bulk JL BioFET, (**d**) ASD-DFSL-Bulk JL-BioFET.

Figure 1 illustrates the device structures of the JL-BioFET employed in the simulation study. The proposed devices operate solely through the drain-source bias without the need for a reference electrode. The active layer thickness is set to 20 nm to ensure a well-defined off-state and achieve high sensitivity. The gate dielectric consists of 2.5 nm of Al_2_O_3_ and 2.5 nm of SiO_2_, which improves key electrical characteristics, such as V_swing_, and enhances sensor stability in bio-liquid environments, thereby ensuring reliable operation [23]. The channel adopts a nanowire architecture with a length of 20 nm and a width of 16 nm, designed to maximize the surface-to-volume ratio and thus improve sensing sensitivity [24]. The detailed specifications of the device used in the simulation are summarized in Table 1.

### 2.2. Physics Models

Multi-physics-based models were utilized to ensure accurate simulation results. Carrier recombination between the active layer and the substrate was described using the Shockley–Read–Hall (SRH) and Auger recombination models [25,26,27]. Band-to-band tunneling (BTBT) effects were incorporated using a nonlocal BTBT model, which captures horizontal tunneling between the channel valence band and the drain conduction band [28]. Carrier mobility degradation due to impurity and carrier–carrier scattering was modeled using the Philips unified mobility model, while the HighFieldSat and Enormal models were implemented to account for high electric field effects on carrier mobility [29]. The band gap narrowing (BGN) effect resulting from heavy doping in the active layer was considered using the OldSlotboom model [30]. For biosensing functionality, a fixed charge trap model was applied to the gate dielectric surface. Following previous reports [31], the biomolecule charge was represented as a fixed charge density of −1 × 10^12^ cm^−2^. Since the focus here is to evaluate the effects of various device design features and compare their impact, this simplified approach is deemed sufficient instead of detailed surface reactions specific to various individual target biomolecules.

**Figure 2:**
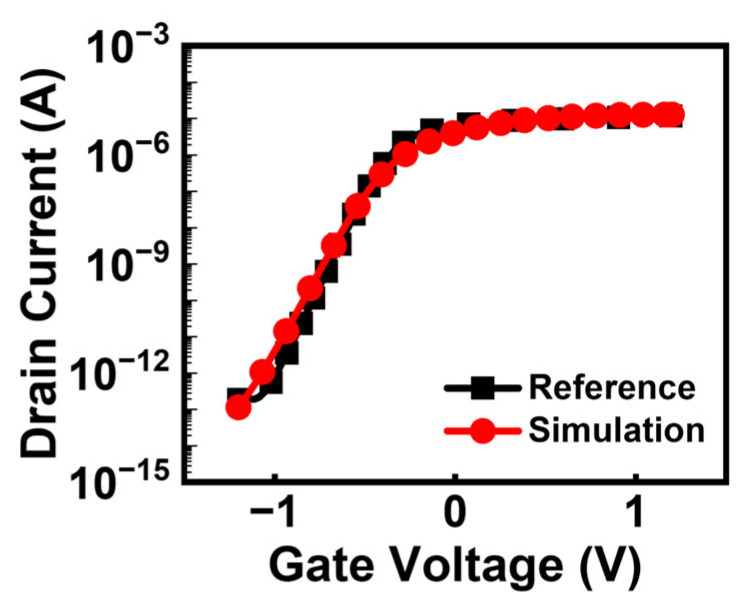
Model calibration results considering tunneling parameters and Shockley-Read-Hall recombination effects.

Figure 2 shows the calibration result using the fabricated junctionless transistor, which features a nanowire width of 15 nm and a height of 20 nm [32]. To achieve consistency with the reference data, the electron and hole tunneling masses in the band-to-band tunneling (BTBT) model were set to 0.4 m_0_ and 0.65 m_0_, respectively. The reference doping concentration (N_ref_) was set at 1 × 10^19^ cm^−3^ to account for SRH recombination effects. Furthermore, the maximum constant carrier lifetime (*τ*_max_) applied in cases without doping dependence was fixed at 2 × 10^−7^ for both electrons and holes. Consequently, when the doping concentration reached 1 × 10^19^ cm^−3^, the corresponding carrier lifetime was determined to be 1 × 10^−7^ s [33]. As seen from Figure 2, the simulation results closely match the measured data reported for the reference device, validating the accuracy of the calibration process.

## 3. Results and Discussion

### 3.1. Operation of JL-BioFET

**Figure 3:**
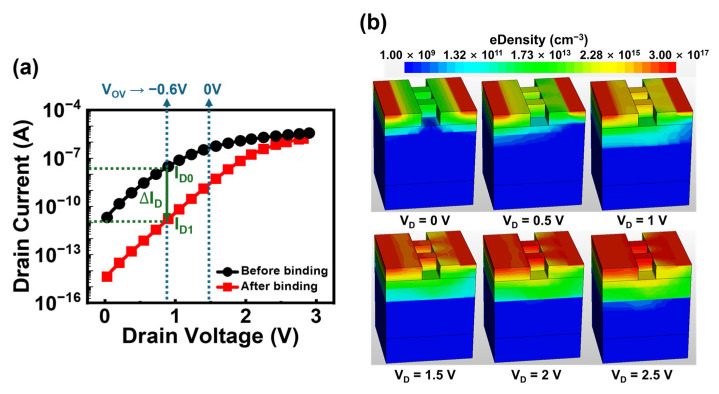
(**a**) I_D_–V_D_ curve of conventional Bulk JL-BioFET and (**b**) electron density distributions in the channel region under varying drain voltages before biomolecule binding.

Figure 3 shows the drain current–drain voltage (I_D_–V_D_) characteristics of a conventional Bulk JL-BioFET. A current-conducting channel is initially formed because the source, drain, and channel region are uniformly doped with the same type of dopant. When the source is grounded and the drain voltage is increased, the drain current initially increases linearly with the drain voltage. This linear behavior is attributed to the presence of a well-formed channel, where the current follows Ohm’s law. As the drain voltage continues to increase, the reverse bias across the p-n junction between the drain and the substrate increases, leading to an expansion of the depletion region. Consequently, the channel thickness near the drain becomes thinner, and the rate of current increase significantly slows down. Eventually, the channel near the drain is fully depleted, resulting in current saturation regardless of further increases in the drain voltage.

Sensitivity is one of the most critical parameters in determining the performance of a biosensing device. The sensitivity of the BioFET is generally quantified using the following definition:

$$Sensitivity=\left(I_{D0}-I_{D1}\right)/I_{D1}$$

The sensitivity is defined as the normalized change in drain current resulting from the binding of biomolecules. Here, I_D0_ denotes the initial drain current of the functionalized device before the attachment of biomolecules, while I_D1_ represents the drain current of the device after the biomolecule binding. The sensitivity was evaluated here at an overdrive voltage (V_OV_ = V_DS_ − V_ON_) of −0.6 V, as shown in Figure 3a. As the overdrive voltage is defined relative to the turn-on condition of each device, it enables a fair performance comparison among devices with different I_D_-V_D_ characteristics. In addition, V_ON_ is defined as the drain voltage obtained by extrapolating the linear portion of the I_D_-V_D_ curve using the linear extrapolation method, considering the absence of a gate voltage. This extrapolation is a commonly used method to extract the threshold voltage of a MOSFET. Similarly, V_swing_ is extracted based on the rate of current change in the region below V_ON_ with respect to the drain voltage. This approach accounts for the channel formation induced by increasing drain voltage and is analogous to the conventional method used for determining V_TH_ and sub-threshold swing (SS) in MOSFETs.

### 3.2. Bulk JL-BioFET with Doped Field Stop Layer

**Figure 4:**
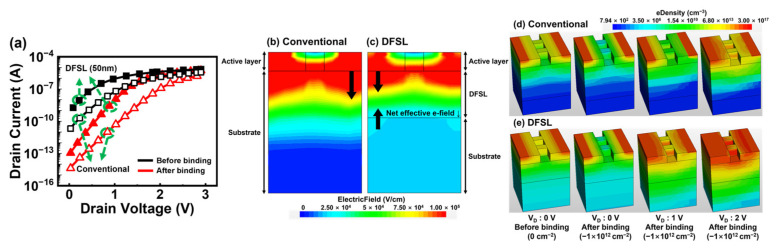
(**a**) I_D_-V_D_ characteristics of two types (conventional and DFSL) of Bulk JL-BioFET, electric field distribution (cross-sectional view) in (**b**) conventional and (**c**) DFSL-Bulk JL-BioFET before biomolecule binding, and electron density in (**d**) conventional and (**e**) DFSL-Bulk JL-BioFET.

Figure 4a presents I_D_-V_D_ characteristics before and after biomolecule binding for two types of devices: one with a 50 nm DFSL and the other with a fully doped substrate with the same doping type (conventional). When the DFSL is applied to the bulk JL-BioFET, the proposed device exhibits enhanced electrical characteristics with a reduced V_ON_ of 1.53 V and an improved V_swing_ of 173 mV/dec. Compared to the conventional device, V_ON_ is reduced by 0.7 V and V_swing_ is improved by 61 mV/dec. In addition, the current variation before and after biomolecule binding is more significant in the DFSL device than in the conventional device, indicating superior sensitivity of the DFSL-based device. The electric field and electron density distributions were examined to analyze the impact of the DFSL on device performance. As shown in Figure 4b, the introduction of the DFSL generates two distinct electric fields: one from the channel toward the DFSL and another from the substrate toward the DFSL. This configuration reduces the net effective electric field from the channel to the substrate, thereby suppressing electron injection from the substrate into the channel compared to the conventional device. As a result, the channel is more effectively controlled by the drain voltage (Figure 4c), leading to improved V_ON_ and V_swing_ characteristics. When negatively charged biomolecules bind to the oxide surface, the electron concentration in the channel decreases due to electrostatic repulsion, leading to a reduction in the drain current. The reduction in substrate-driven electron compensation enhances the variation in channel electron density upon biomolecule attachment, thereby enhancing the modulation of channel electron density upon biomolecule attachment and significantly improving the device’s sensitivity. In contrast, the conventional device shows an electric field formed from the active layer toward the substrate at their interface. This field induces electron injection from the substrate into the channel, reducing the channel’s responsiveness to changes in drain voltage (Figure 4c) and leading to large V_ON_ and V_swing_. In conventional devices, the electric field induces electron injection from the substrate into the channel, which reduces the change in channel electron concentration caused by the biomolecule binding. As a result, the current variation in response to biomolecule attachment is reduced, leading to a degradation in sensitivity.

**Figure 5:**
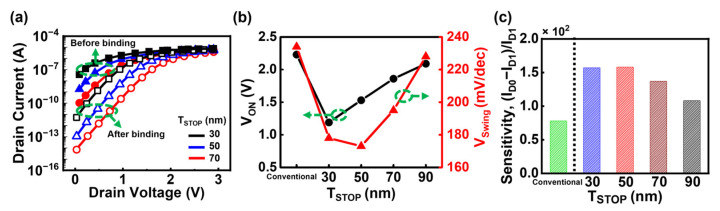
Effect of T_STOP_ on the performance of DFSL Bulk JL-BioFET: (**a**) I_D_-V_D_ characteristics, (**b**) V_ON_ and V_swing_, and (**c**) sensitivity.

Figure 5a–c shows the simulation results of DSFL-Bulk JL-BioFET with varying DFSL thickness (T_STOP_) values. In Figure 5a, the drain current at 0 V is defined as the off current, which decreases as T_STOP_ increases. This trend is attributed to the expansion of the depletion region between the active layer and the DFSL as the DFSL becomes thicker, thereby reducing the effective channel thickness within the active layer. Given that the doping concentrations of the active region and the DFSL are 3 × 10^17^ cm^−3^ and 1.5 × 10^17^ cm^−3^ respectively, the theoretical depletion width is approximately 100 nm. However, both the active layer and the DFSL are thinner than this value, so increasing the DFSL thickness effectively extends the depletion region. As the DFSL thickness decreases, the channel controllability improves, leading to a lower V_ON_ and improved V_swing_, as shown in Figure 5b. Furthermore, the sensitivity, which reflects the biosensing performance, is also improved (Figure 5c). When the DFSL thickness is 50 nm, the device achieves the best V_swing_ of 173 mV/dec and exhibits the highest sensitivity, approximately twice that of the conventional Bulk JL-BioFET. These findings indicate that a DFSL thickness of 50 nm provides optimal biosensing performance. Conversely, if the active layer is excessively thin or thick, the modulation of the channel conductivity by biomolecule binding becomes less effective, resulting in degraded sensing performance.

### 3.3. DFSL Bulk JL-BioFET with Asymmetric Source/Drain Structure

**Figure 6:**
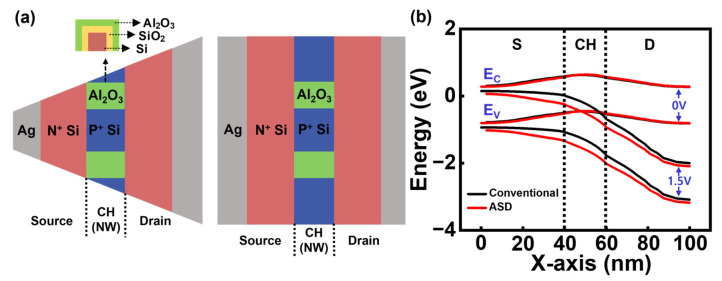
(**a**) Top view schematic and (**b**) energy band diagram of the conventional Bulk JL-BioFET with asymmetric and symmetric source/drain (ASD) structures.

Figure 6a shows the top view schematic of the Bulk JL-BioFET with asymmetric source/drain (ASD) structure along with its symmetric counterpart. The source and drain electrode regions in the symmetric structure are designed to have the same dimensions. In contrast, the source electrode region in the asymmetric structure is designed such that its top-view area decreases with increasing distance from the channel, while the drain electrode region is designed so that its top-view area increases with distance from the channel. Figure 6b shows the energy band diagrams under drain bias conditions of 0 V and 1.5 V. According to the band diagrams, the asymmetric source/drain structure exhibits a steeper energy variation between the source and drain compared to the symmetric structure, with this effect being particularly pronounced near the source region. This difference in energy band variation implies corresponding differences in the voltage distribution and electric field profile. Specifically, the potential gradient at the source–channel junction under the same applied drain voltage is steeper in the asymmetric structure than in the symmetric structure, leading to a stronger electric field at the source–channel junction. The increased electric field facilitates electron injection from the source into the channel and enhances electron velocity. Consequently, this can lead to an improvement in V_swing_ and a reduction in V_ON_, thereby enhancing the overall electrical performance of the device.

### 3.4. Comparison of the Sensitivity of Proposed Structures

**Figure 7:**
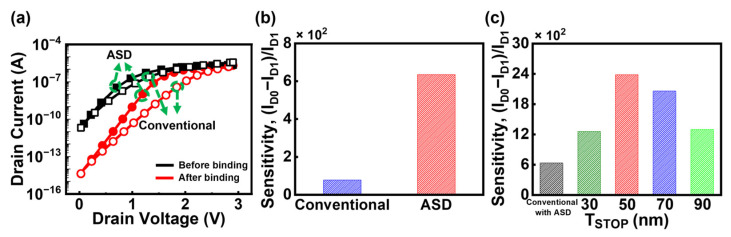
(**a**) I_D_-V_D_ characteristics and (**b**) sensitivity of two types (asymmetric and symmetric source/drain structures) Bulk JL-BioFET. (**c**) Effect of T_STOP_ on the sensitivity of ASD-DFSL-Bulk JL-BioFET.

Figure 7a shows the I_D_-V_D_ curves of ASD-Bulk JL-BioFET before and after biomolecule binding. The device incorporating the ASD structure demonstrates enhanced electrical performance compared to the symmetric structure with a reduced V_ON_ of 1.45 V (a decrease of 0.78 V compared with the symmetric structure) and an improved V_swing_ of 184 mV/dec (a decrease of 50 mV/dec compared with the symmetric structure). As analyzed in Figure 6b, these improvements are attributed to the increased electric field near the source region resulting from the asymmetric geometry. The sensitivity of the device was also analyzed to evaluate the biosensing capability, as shown in Figure 7b. The device with the ASD structure exhibits a sensitivity of 635, which is approximately 8 times higher than that of the conventional Bulk JL-BioFET. This significant enhancement in sensitivity is a combined effect of the DFSL and ASD structures. Figure 7c illustrates the influence of DFSL thickness on the sensitivity. The sensitivity reaches a maximum of 2380 at T_STOP_ of 50 nm, which is about 3.7 times higher than that of the conventional device with ASD structure. These results, consistent with earlier analyses, confirm that the DFSL effectively enhances channel controllability and significantly enhances biosensing sensitivity.

**Figure 8:**
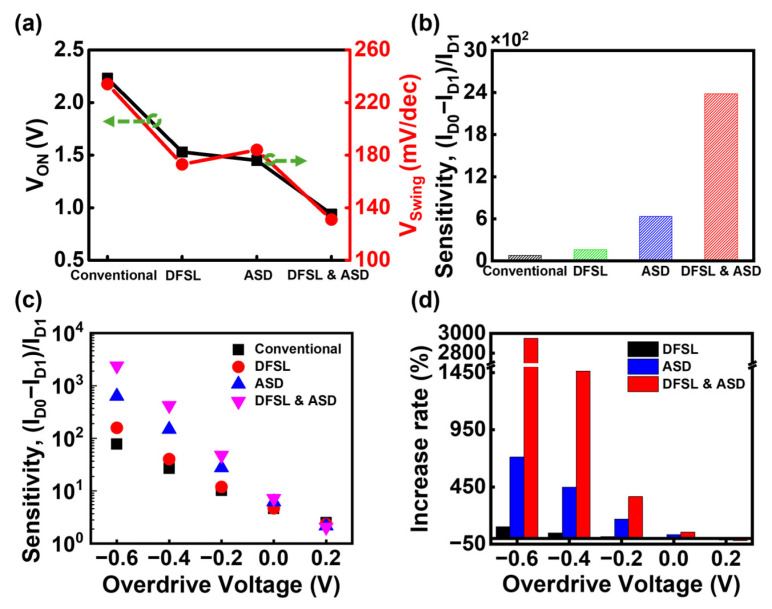
Performance comparison between four types of Bulk JL-BioFET: (**a**) electrical parameters, (**b**) sensitivity, (**c**) overdrive voltage V_OV_-dependent sensitivity, and (**d**) increase rate of sensitivity compared to conventional Bulk JL-BioFET.

Figure 8 shows the performance comparison among four types of JL-BioFETs. Compared to the conventional device, those incorporating either DFSL or ASD demonstrate enhanced electrical and biosensing characteristics. The device integrating both DFSL and ASD exhibits the lowest V_ON_ and V_swing_ values, leading to the highest sensitivity. In this configuration, the sensitivity is approximately 30 times higher than that of the conventional device without DFSL and ASD. The combination of the DFSL and ASD structure plays a key role in optimizing the electrical and biosensing characteristics. The ASD structure is found to be slightly more effective than the DFSL in enhancing Bulk JL-BioFET performance. Figure 8c shows the sensitivity as a function of overdrive voltage V_OV_. When V_OV_ is above 0 V, the sensitivity of the device with both DFSL and ASD structures does not show a significant improvement compared to other devices. However, the sensitivity enhancement becomes most pronounced when V_OV_ falls below 0 V. At V_OV_ = −0.6 V, the sensitivity increases by up to approximately 3000% compared to the conventional device. These results indicate that optimizing the operating region is crucial to maximizing sensitivity even in devices combining DFSL and ASD. Operating the device in the V_D_ range below the V_ON_ leads to a significant improvement in sensitivity.

## 4. Conclusions

We proposed a novel Bulk JL-BioFET design incorporating a doped field stop layer (DFSL) and an asymmetric source/drain (ASD) structure to enhance both electrical and biosensing performance. The electrical characteristics and sensing performance were analyzed by TCAD simulations. The introduction of the DFSL was shown to improve device performance by suppressing electric field penetration into the substrate, thereby enhancing channel controllability. As a result, the device exhibited a reduction in turn-on voltage (V_ON_) by 0.7 V and an improvement in the on-off swing voltage (V_swing_) by 61 mV/dec compared to the conventional structure. Furthermore, sensitivity analysis with varying DFSL thickness (T_STOP_) values revealed that the optimal value was achieved at 50 nm, yielding the highest sensitivity. The effects of the ASD structure were also investigated. The ASD configuration significantly enhanced V_ON_, V_swing_, and sensitivity. These improvements are attributed to the enhanced electric field at the source–channel junction, which promotes efficient electron injection and higher carrier velocity. When both the DFSL and ASD structures were applied, the device demonstrated a sensitivity approximately 30 times greater than that of the conventional Bulk JL-BioFET. These results highlight the synergistic effect of the DFSL and ASD structures in maximizing biosensor performance through improved channel controllability. Finally, we investigated the sensitivity variation according to the operating bias and found that the sensitivity is maximized when the device operates below the V_ON_. The sensitivity was improved by up to 3000% in this bias region, compared to the conventional device. These findings confirm that the integration of the DFSL and ASD structures plays a critical role in enhancing the performance of the Bulk JL-BioFET. Moreover, the results highlight the importance of optimizing the operating bias to fully exploit the advantages of the proposed design for high-performance biosensing applications.

The proposed DFSL and ASD features are well within the modern integrated circuit fabrication capabilities. Both features can be readily implemented with standard CMOS processes, including lithography, ion implantation, etching, etc., as the proposed sensor is based on a conventional silicon device without requiring special materials or complex steps. In practice, the main concerns would be achieving stable control of the DFSL doping profile and keeping the dielectric/channel interface free from excessive traps. These factors may cause small differences compared with the simulation results, such as a shift in threshold voltage, a lower on/off ratio, or variations in sensitivity. Even so, such effects are typical process variations, and the advantages of the DFSL and ASD features are expected to remain in fabricated devices. Finally, the absence of an external reference electrode provides a practical advantage for large-scale integration, since it removes the need for bulky reference electrodes often used in BioFETs and allows sensor arrays to be implemented directly on-chip at the wafer level. Therefore, the device concept is well suited for development into practical high-density biosensor platforms.

## Figures and Tables

**Table 1:** Simulation parameters for the proposed device.

Description (Parameter)	Values
Active layer (T_AL_)	20 nm
SiO_2_ thickness (T_SiO_2__)	2.5 nm
Al_2_O_3_ thickness (T_Al_2_O_3__)	2.5 nm
Source region length (L_Source_)	40 nm
Drain region length (L_Drain_)	40 nm
Nanowire channel length (L_NW_)	20 nm
Nanowire channel width (W_NW_)	16 nm
N^+^-type Si doping concentration (C_N_+)	3 × 10^17^ cm^−3^
P^+^-type Si doping concentration (C_P_+)	1.5 × 10^17^ cm^−3^
P-type Si doping concentration (C_P_)	1 × 10^15^ cm^−3^

## Data Availability

The data is contained within the manuscript.
